# Engaging men to promote and support exclusive breastfeeding: a descriptive review of 28 projects in 20 low- and middle-income countries from 2003 to 2013

**DOI:** 10.1186/s41043-017-0127-8

**Published:** 2017-12-15

**Authors:** Jennifer M. Yourkavitch, Jeniece L. Alvey, Debra M. Prosnitz, James C. Thomas

**Affiliations:** 1 Department of Epidemiology, Gillings School of Global Public Health, University of North Carolina at Chapel Hill, 135 Dauer Drive, 2101 McGavran-Greenberg Hall, CB #7435, Chapel Hill, NC 27599-7435 USA; 2 Public Health Institute/Global Health Fellows Program II, Washington, DC, USA; 3 Division of International Health and Development ICF, Rockville, MD USA

**Keywords:** Exclusive breastfeeding, Male involvement, Gender

## Abstract

**Background:**

Lay support has been associated with improved breastfeeding practices, but studies of programs that engage men in breastfeeding support have shown mixed results and most are from high-income countries. The purpose of our research is to review strategies to engage men in exclusive breastfeeding (EBF) promotion or support in 28 project areas across 20 low- and middle-income countries. This information may be used to inform program implementers and policymakers seeking to increase EBF.

**Methods:**

We tested the difference between baseline and final EBF proportions using Pearson’s chi-square (*a* = 0.05) and identified project areas with a significant increase. We categorized male engagement strategies as low- and high-intensity, using information from project reports. We looked for patterns by intensity and geography and described strategies used to engage men in different places.

**Results:**

Twenty-eight projects were reviewed; 21 (75%) were in areas where a statistically significant increase in EBF was observed between the beginning and end of the project. A variety of high- and low-intensity male engagement strategies was used in areas with an increase in EBF prevalence and in all geographic regions. High-intensity strategies engaged men directly during home or health visits by forming men’s groups and by working with male community leaders or members to promote EBF. Low-intensity strategies included large community meetings that included men, and radio messages, and other behavior change materials directed towards men.

**Conclusion:**

Male engagement strategies took many forms in these project areas. We did not find consistent associations between the intensities or types of male engagement strategies and increases in EBF proportions. There is a gap in understanding how gender norms might impact male involvement in women’s health behaviors. This review does not support the broad application of male engagement to improve EBF practices, and we recommend considering local gender norms when designing programs to support women to EBF.

## Background

Gender factors affect maternal and child health in many ways and often manifest in terms of gender inequality through control of resources, decision-making, and access to health information, which can affect behaviors that in turn affect the mother’s and her child’s health [1]. The relationship of breastfeeding with gender equality raises the question of whether breastfeeding is solely the responsibility of the mother [2]. Exclusive breastfeeding (EBF) has been shown to provide immediate and long-term benefits for both mothers and children [3–7]. Both skilled and lay (e.g., peer) support have been shown to reduce the risk of suboptimal breastfeeding practices [8, 9] with face-to-face support being the most effective for EBF [8], but effective approaches and strategies to support in different geographic, cultural, and income contexts are still being studied.

A key constraint to EBF in some lower- and middle-income countries (LMIC) is lack of knowledge and support from household members who wield authority over many household practices, including infant feeding decisions, particularly fathers and grandmothers [10–13]. There have been many social and behavior change efforts to engage men in reproductive health program interventions, but evidence regarding the impact on breastfeeding practice is mixed [1, 14]. Moreover, many of the studies about engaging men in EBF promotion and support were in higher-income countries and are thus of unknown relevance to LMIC (e.g., [15–20]).

The few studies conducted in LMIC showed positive EBF outcomes when men were included in interventions. An intervention in Vietnam providing fathers with breastfeeding education materials, counseling, and household visits found significantly higher EBF at 4 and 6 months, compared to mothers whose partners were not in the intervention group [21]. Similarly, an intervention in Turkey found that EBF prevalence was highest in a group where both mothers and fathers received breastfeeding education, compared to a group where only mothers received it [22]. A clinical trial in Brazil found that EBF increased where fathers were included in a breastfeeding education program [23].

Since 1985, the United States Agency for International Development’s (USAID) Child Survival and Health Grants Program (CSHGP) has supported nongovernmental organizations’ (NGO) efforts to reduce maternal and child morbidity and mortality through interventions designed to address health issues, including EBF. USAID provides technical assistance to NGOs in designing, implementing, monitoring, and evaluating these projects, and maintains a database for project information.

The purpose of our research was to review strategies to engage men in supporting the practice EBF by their partner in 28 CSHGP project areas in 20 LMIC. This information may inform program implementers and policymakers seeking to increase EBF practices. Documenting and disseminating results from community-based programs in a variety of country contexts can inform strategies to reach global goals to reduce preventable child deaths.

## Methods

We included all CSHGP projects beginning and ending between 2003 and 2013 that (1) reported engaging men in EBF support, (2) were conducted in LMIC in sub-Saharan Africa, Southeast Asia, South and Central Asia, or Latin America/Caribbean regions, and (3) had complete survey data. We considered survey data complete if the questions included the number of infants under 6 months of age who were exclusively breastfed in the previous 24 h, and the total number of infants under 6 months of age in the survey, with no indication of data quality concerns in the final evaluation report. The University of North Carolina’s Institutional Review Board (IRB) determined that this study was not human subject research and does not require IRB approval. It is an analysis of secondary data (project reports) with no personal identifiers.

### Quantitative methods

Each NGO conducted population-based knowledge, practice, and coverage surveys in their project areas at the beginning and end of their projects. These surveys were designed and conducted with assistance from USAID and employed a standardized methodology, including obtaining informed consent and data collection instruments designed by survey sampling and design experts [24]. NGOs used either 30-cluster or Lot Quality Assurance sampling methodologies to obtain project area prevalence estimates for EBF. With permission from the USAID, we extracted data from the CSHGP database and project reports. The standard calculation of EBF prevalence is the number of infants under 6 months of age who were given only breast milk in the 24 h preceding the survey divided by the number of infants under 6 months of age surveyed, times 100. We calculated Pearson chi-square statistics to test the association between time (baseline or final) and EBF using SAS (version 9.4, Cary, NC). That test indicates if the proportions are statistically different at *a* = 0.05.

### Qualitative methods

We extracted qualitative information about EBF promotion and support strategies from project reports and thematically analyzed it to aid interpretation of our results. Our primary search terms included men, husband(s), father(s), exclusive, breastfeeding, and LAM (lactational amenorrhea method—a modern contraceptive method that requires full breastfeeding as one of the criteria for use). If searching for men, husband(s), and father(s) yielded no results, we searched for family, partner, male, and decision-maker. Upon identification of keywords in the text, we reviewed the report and any relevant annexes to ascertain the strategies used to engage men. We populated a matrix with information extracted from the reports related to male roles, EBF promotion or support activities that engaged men, activity frequency and timing, intensity of male engagement strategy, as well as quotes about male engagement or lack of male engagement, other EBF promotion or support activities, and other project health activities that did not engage men. We then organized male engagement activities by intensity and geographic region. We categorized male engagement strategy into three levels by intensity: none, low, and high. “Low” included indirect male engagement strategies such as general mass outreach efforts such as community health fairs and mass media campaigns; “high” included male engagement strategies with direct personal contact, such as interpersonal communication through home visits or through groups such as community-based organizations.

## Results

**Fig. 1:**
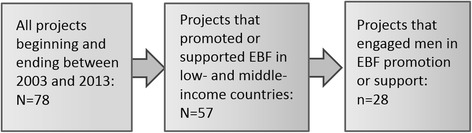
Flowchart for project selection

**Fig. 2:**
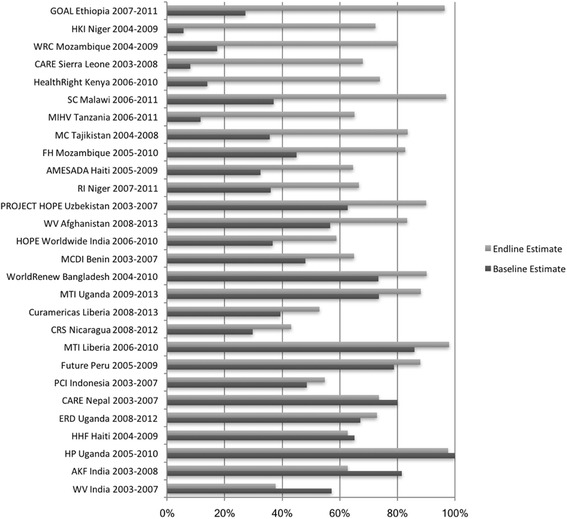
Exclusive breastfeeding prevalence estimates at beginning and end of projects

Twenty-eight projects that reported strategies to engage men to promote or support EBF were included (Fig. 1). Fifteen projects were implemented in sub-Saharan Africa, eight in South or Central Asia, four in the Latin America/Caribbean region, and one in Southeast Asia. Twenty-one project areas (75%) had significant increases in EBF prevalence at the end of project implementation (Table 1; Fig. 2).

**Table 1:** Project characteristics^a^

	NGO^b^	Location	Years	Population	Baseline EBF prevalence (95% CI)	Final EBF prevalence (95% CI)	Region	Intensity of male engagement
1.	AKF	India	2003–2008	88,128	80.1 (75.4–84.8)	62.9 (51.2–74.6)*	SCA	High
2.	AME-Sada	Haiti	2005–2009	300,000	32.4 (15.6–49.2)	64.8 (46.9–82.7)*	LAC	Low
3.	CARE	Nepal	2003–2007	931,054	66.8 (54.6–79.0)	73.5 (66.6–80.4)	SCA	Low
4.	CARE	Sierra Leone	2003–2008	112,921	8.3 (1.6–15.0)	68.4 (57.5–79.3)*	SSA	Low
5.	CRS	Nicaragua	2008–2012	113,560	29.7 (23.0–36.4)	43.2 (35.1–51.3)*	LAC	High
6.	Curamericas	Liberia	2008–2013	149,322	39.4 (26.1–52.7)	52.9 (39.2–66.6)*	SSA	Low
7.	ERD	Uganda	2008–2012	53,083	67.1 (60.8–73.4)	73.0 (67.0–79.0)	SSA	High
8.	FH	Mozambique	2005–2010	254,282	40.0 (31.0–49.0)	81.5 (73.8–89.2)*	SSA	High
9.	FG	Peru	2005–2009	119,478	79.0 (65.7–92.3)	87.9 (73.1–100.0)^c^*	LAC	High
10.	GOAL	Ethiopia	2007–2011	168,636	27.2 (19.0–35.4)	96.5 (93.1–99.9)*	SSA	High
11.	HealthRight	Kenya	2006–2010	257,083	13.8 (8.3–19.3)	73.7 (64.8–82.6)*	SSA	High
12.	HHF	Haiti	2004–2009	171,703	65.1 (54.8–75.4)	62.8 (52.6–73.0)	LAC	High
13.	HP	Uganda	2005–2010	759,201	100.0 (0)	97.6 (92.9–100.0)^c^	SSA	Low
14.	HKI	Niger	2004–2009	359,400	5.7 (0–12.6)^c^	72.4 (57.1–87.7)*	SSA	High
15.	HW	India	2006–2010	211,070	36.7 (21.4–52.0)	58.9 (48.6–69.2)*	SCA	High
16.	MC	Tajikistan	2004–2008	204,448	35.6 (26.3–44.9)	83.5 (72.7–94.3)*	SCA	High
17.	MCDI	Benin	2003–2007	146,210	48.0 (32.0–64.0)	64.9 (54.2–75.6)*	SSA	High
18.	MTI	Liberia	2006–2010	127,124	86.0 (68.4–100.0)^c^	98.0 (78.5–100.0)^c^*	SSA	High
19.	MTI	Uganda	2009–2013	113,400	73.6 (47.6–99.6)	88.2 (78.9–97.5)*	SSA	High
20.	PCI	Indonesia	2003–2007	76,549	48.5 (36.4–60.6)	54.8 (39.7–69.9)	SEA	High
21.	Project HOPE	Uzbekistan	2006–2011	315,962	62.7 (49.9–75.5)	90.0 (84.5–95.5)*	SCA	High
22.	RI	Niger	2007–2011	454,869	36.1 (19.9–52.3)	66.7 (53.9–79.5)*	SSA	Low
23.	SC	Malawi	2006–2011	724,873	36.6 (24.0–49.2)	96.7 (90.4–100.0)^c^*	SSA	High
24.	WI	Tanzania	2006–2011	218,654	11.6 (2.0–21.2)	65.1 (52.3–77.9)*	SSA	Low
25.	WRC	Mozambique	2004–2009	227,260	17.4 (6.8–28.0)	80.0 (68.0–92.0)*	SSA	High
26.	WR	Bangladesh	2004–2010	169,803	74.2 (66.4–82.0)	90.1 (85.9–94.3)*	SCA	High
27.	WV	Afghanistan	2008–2013	260,500	56.7 (42.6–70.8)	83.5 (74.6–92.4)*	SCA	High
28.	WV	India	2003–2007	3,254,203	57.2 (48.9–60.5)	37.7 (34.5–40.9)*	SCA	Low

*Abbreviations*: *NGO* nongovernmental organization, *CI* confidence interval, *SSA* sub-Saharan Africa, *AKF* Aga Khan Foundation, *SCA* South and Central Asia, *AME-Sada* African Methodist Episcopal Church Service and Development Agency, *LAC* Latin America and Caribbean, *ARC* American Red Cross, *SEA* Southeast Asia, *CHS* Center for Human Services, *CW* Concern Worldwide, *CI* Counterpart International, *CRS* Catholic Relief Services, *DRC* Democratic Republic of Congo, *ERD* Episcopal Relief and Development, *FH* Food for the Hungry, *FG* Future Generations, *HAI* Health Alliance International, *HHF* Haitian Health Foundation, *HP* Health Partners, *HKI* Helen Keller International, *HW* Hope Worldwide, *IRD* International Relief and Development, *MC* Mercy Corps, *MCDI* Medical Care Development Inc., *MTI* Medical Teams International, *PCI* Project Concern International, *Plan* Plan International, *RI* Relief International, *SAWSO* Salvation Army World Service Organization, *SC* Save the Children, *WI* Wellshare International, *WR* World Relief, *WR* World Renew, *WV* World Vision

*Statistically significant (*a* = 0.05) difference in proportions (*n* = 23)

^a^As reported by grantees to USAID

^b^All projects are implemented in partnership with local health service providers, organizations or institutes

^c^The confidence interval was truncated at the extreme value because the margin of error rendered an improbable confidence limit

High-intensity male engagement strategies were reported by 20 projects (Table 2). Sixteen (80%) of the 20 projects with high-intensity male engagement strategies were in areas where EBF prevalence significantly increased. The strategies included working within men’s groups (HealthRight, Kenya) and farmer development associations (Aga Khan Foundation, India and World Renew, Bangladesh); trained community members to promote EBF (Food for the Hungry, Mozambique); included men in-home visits (GOAL, Ethiopia and Mercy Corps, Tajikistan); implemented educational modules in the primary health centers specifically for couples (Project Hope, Uzbekistan), or created breastfeeding support groups for men (Helen Keller International, Niger). Some of these strategies were included in the project objectives from the planning stages, for example, as part of the project equity strategy (Helen Keller, Niger), while others were implemented after midterm evaluation findings indicated the need to engage men, considering women’s lack of decision-making authority in households (HealthRight, Kenya). There were no discernable geographic patterns in terms of the form of male engagement. Four projects with high-intensity strategies did not record an increase in EBF prevalence, and most of them (ERD, Uganda; AKF, India; and HHF, Haiti) provided health education to groups, such as farmers’ groups and fathers’ groups.

**Table 2:** Descriptions of strategies utilized promote and support EBF, grouped by intensity of male engagement strategy and region

NGO, country	Male engagement strategy	Other strategies
High intensity strategies to engage men in EBF promotion or support		
Sub-Saharan Africa		
ERD, Uganda	Community-based organizations (CBOs) such as literacy groups and farmers groups formed and discussed maternal and child health and nutrition topics^a^; household visits to promote behavior change communication messages, including EBF^b^	Village health teams provided breastfeeding advice and information
FH, Mozambique^*^	Care Group model, with the majority of community-selected promoters^b^ male (85%)	
GOAL, Ethiopia^*^	Community-level promotion of Community Integrated Management of Childhood Illness (CIMCI) and Maternal, Neonatal, and Child Health and Nutrition (MNCH/N); conducted home visits using Care Group Model^a^	
HealthRight, Kenya^*^	Monthly meetings held with male dominated CBOs and Faith-based Organizations (FBOs) for health topic discussions and dissemination of behavior change communication (BCC) materials with community health workers (CHWs)^a^; home visits conducted by CHW for maternal and newborn health education entire family^b^	Participated in week-long national promotion campaigns
HKI, Niger^c*^	Breastfeeding Support Groups that included men and women^b^; created community-based growth promotion teams including at least 1 man to disseminate Essential Nutrition Actions (ENA) messages (including EBF) at community events^b^	ENA committees also promoted EBF
MCDI, Benin^c*^	Targeted EBF behavior change information, education communication (IEC) materials, and radio spot messages towards fathers as household decision-makers^b^; men participated in community song festivals and radio contests with key breastfeeding messages^b^	BCC and IEC materials for mothers, including radio spots, integrating matrons and mothers-in-law in breastfeeding promotion, VISA (leader) mothers and CHWs promoted messages and were trusted by the community
MTI, Liberia^c*^	Household Health Promoters provided home visits and community education sessions^b^	Coordinated support for infant and young child feeding at community and facility levels.
MTI, Uganda^*^	Community-identified men trained as members of Village Health Teams to deliver health messages through community mobilization activities and IEC materials for project intervention areas^b^; men trained as peer educators to deliver weekly early child development modules to parents^b^	
SC, Malawi^c*^	Village Health Committees mobilized “core groups” of women and men to identify barriers to recommended practices and implement local activities related to newborn health^b^; trained grandparents, including grandfathers, to give counseling and deliver health education messages on key maternal and newborn health topics, including essential newborn care^b^	Home visits to pregnant and postpartum women
WR, Mozambique^*^	Formed Care Groups with Pastors/Traditional Healers to share health messages with the community^a^	
South and Central Asia		
AKF, India^c*^	Health education in CBO meetings (e.g., Farmers Groups)^a^	
HW, India^*^	Trained Community Health Teams provided individual family or small group counseling from for fathers, mothers, pregnant women, etc.^b^; engaged religious leaders to communicate healthy behavior messages	
MC, Tajikistan^*^	Trained Community Health Educators and Village Development Committees (composed of local men and women) worked at community level by focusing behavior change and nutrition messaging towards household decision-makers (men and mothers-in-law)^b^	Mothers’ Groups/Breastfeeding Support Groups; support for district maternity houses to gain or renew Baby-Friendly status
Project HOPE, Uzbekistan^c*^	Trained community leaders to deliver health messages (including EBF) to families during household visits and community events^b^; created New Parents’ Schools in community health centers to educate expectant parents on health topics such as breastfeeding^b^	Assisted hospitals to gain Baby-Friendly certification; breastfeeding support groups at maternity houses; participation in annual Breastfeeding Week activities; monitoring Baby-Friendly policy adherence at maternity houses; dissemination of breastfeeding educational materials
WR, Bangladesh^*^	Used community based organization to form primary groups of men, including husbands and community leaders, to promote key family practices critical for child health and nutrition^a^	
WV, Afghanistan^*^	Formed community-level committees (shuras) to mobilize communities and health shura members to communicate messages from Home-based Life Saving Skills (HBLSS)^b^; conducted timed and targeted counseling home visits for pregnant women, other caregivers, and household decision-makers^b^; held community meetings for promoting HBLSS messages^b^	Promoted and supported Baby-Friendly Hospital Initiative; women peer groups
Southeast Asia		
PCI, Indonesia	Community outreach and counseling events for parents and caregivers^b^	
Latin American and the Caribbean		
CRS, Nicaragua^c*^	Behavior Change Agents using religious gatherings and sporting events to promote BCC strategies; specific program Engaging Men to Improve Care-Seeking; TBA home visits with women and partner to promote health topics, including EBF^b^	Strengthening health workers’ and volunteers’ capacities related to maternal and newborn nutrition
FG, Peru^*^	Community Health Agent home visits geared towards families^b^; general community assemblies discussing health issues of women and children^b^	Integrated with other health messages, e.g., EBF to prevent pneumonia; trained health facility staff and community health agents
HHF, Haiti	Organization of Fathers’ Groups for health education activities^a^; community meetings and demonstrations^b^	
Low-intensity strategies to engage men in EBF promotion or support		
Sub-Saharan Africa		
Care, Sierra Leone^c*^	Formed community health clubs, with concerted effort to include men, and promoted health messages at meetings^b^	Trained community-based growth promoters to promote EBF; pregnant women’s support groups and multisectoral activities promoted nutrition behaviors
Curamericas, Liberia^c*^	Behavior change communication activities in communities with messages targeted at both genders^b^	
HP, Uganda^c^	Conducted community BCC sessions promoting breastfeeding^b^	Counseled mothers on breastfeeding; behavior change communication activities with men
RI, Niger^*^	Conducted meetings with husbands and village committees to promote behavior change communication messages, which include breastfeeding^a^	Promoted health behaviors with women’s health groups
WI, Tanzania^*^	Embedded EBF messages into other BCC message health topic areas, including diarrhea and pneumonia, at community events^b^	
South and Central Asia		
Care, Nepal^c^	Behavior change communication strategy targeted husbands, including radio, TV and other IEC materials disseminated at community events^b^	Trained Female Community Health Volunteers to educate and counsel mothers
WV, India^c*^	Held community meetings to improve men’s engagement in family planning (especially LAM) and maternal and child nutrition	Timed counseling sessions with mothers; CHW training
Latin American and the Caribbean		
AME-Sada, Haiti^*^	Organized community-wide rally posts to educate community, including fathers, in-laws, and grandmothers, to communicate specific behavior change messages, including EBF^b^	Trained CHWs, who made home visits; partnered with COZAM (breastfeeding promotion group); behavior change messages communicated through several media, including breastfeeding clubs and support groups

^*^Statistically significant (*a* = 0.05) difference in proportions (*n* = 23)

^a^Engaged women in similar but separate activities as men for EBF promotion and support

^b^Engaged women alongside men in same activities for EBF promotion and support

^c^Conducted formative research to inform strategies to engage men in EBF promotion and support; includes qualitative methods such as focus group discussion, barrier analysis, doer/nondoer analysis, or other surveys

Low-intensity male engagement strategies were used by the eight projects across nearly all regions and included large community meetings (Relief International, Niger; AME-Sada, Haiti, and World Vision, India), radio messages (Care, Sierra Leone), or other behavior change messaging and materials (Care, Nepal; Curamericas, Liberia; Wellshare International, Tanzania; and HealthPartners, Uganda). Six (75%) of the projects reporting low-intensity strategies to engage men worked where EBF prevalence significantly increased. One such project used a combination of low-intensity strategies with high frequency, including monthly community meetings and other behavior change communication materials (Care, Sierra Leone).

Eleven project reports also documented other strategies used to promote and support breastfeeding. These other strategies included training other influencers, such as mothers-in-law, strengthening community health workers’ capacities, and promoting behavior change in communities with various messages and media.

## Discussion

This study reviewed projects that employed strategies to engage men in EBF promotion and support in several geographic settings and calculated EBF prevalence changes in project areas. No clear pattern emerged, in terms of increased EBF prevalence where certain strategies were employed or in the choice of strategies in different geographic areas. In addition, not every project area had an increase in EBF prevalence.

We do not know how effective the reported male engagement strategies were in engaging men because this type of analysis was not conducted as part of the project evaluations. Several independent project evaluators commented on male engagement, or lack of male engagement, in their final evaluation reports, with most indicating approval of male engagement to promote or support EBF. Only one comment considered having male volunteers speak with mothers about breastfeeding techniques as potentially inappropriate (American Red Cross, Cambodia). The final evaluator for Care’s project in Sierra Leone noted that the concerted effort during project planning to include household decision-makers (men and older women) in the project approaches contributed to the adoption of positive health behaviors among women, which was revealed by women in project focus group discussions.

With increased emphasis on male involvement in the reproductive health care and decisions in global health, it is important to understand where engaging men as a social and behavior change approach, broadly speaking, may support EBF practice and if it could hinder it. This study provided a mainly descriptive review of strategies, and we conclude that, unlike peer support, professional support in the antenatal and postpartum periods, and other evidence-based strategies [8], engaging men in EBF promotion and support has had mixed results and appears to be highly dependent on context. Thus, it cannot be assumed to be appropriate or effective everywhere. There is evidence of its success in some contexts, both high- (e.g., [15]) and low-income (e.g., [21]), but local gender factors related to decision-making, power, autonomy, and what is considered “women’s space” should be understood before “engaging men” is adopted as a general approach. In addition, most projects that reported a male engagement strategy also reported engaging women (mothers and grandmothers) alongside men (Table 2), making it difficult to disentangle the approaches. We do not know if strategies to engage men alone would have had a different impact on EBF prevalence in these areas.

One cannot consider male engagement in women’s health issues without considering the gender norms that govern relationships in households and communities. Less than one-quarter of these projects reported using formative research to inform their strategies (Table 2). Formative research would enable the opportunity to investigate gender norms in order to create an appropriate intervention that is gender-sensitive and could, in some circumstances, be gender transformative [25]. This type of formative research could also inform a project-wide gender and social and behavior change strategy. Whereas decisions about health care often involve money for travel and services, and money is often under the jurisdiction of male authority, decisions about infant care are often left to mothers themselves. Some societies have deeply embedded cultural beliefs about the gendered division of family responsibilities, with men focusing on financial matters and women focusing on household matters, even when those women work in the formal sector or outside of the home, as documented by Nkwake [26] in Uganda. Taking care of the family, deference to men, and inequality with men at home and in public are gender norms that form a “model of domestic virtue,” which has persisted over the past century [27]. Likewise, in Benin, there is evidence of persistent gender disparities regarding access to and control of resources, and men often make decisions related to health care [28]. Where women’s movements are restricted or require male permission, as documented in Liberia, Sierra Leone, Nepal, Bangladesh, and Afghanistan [29–33], they may not be able to access or provide peer breastfeeding support, which has been shown to reduce suboptimal breastfeeding practices [8].

The impact of gender norms on women’s infant feeding practices is not well understood. Meanwhile, there is some evidence that high rates of child stunting (low height for age) can co-exist with relatively high values for positive indicators of child health, such as immunization coverage [34]. Stunting reflects both mothers’ and children’s health status; where women have little autonomy, are deprived of their rights to education and health, and are forced into early marriage, their health is negatively affected [35–37], and thus, the health of their children is negatively affected.

There is evidence of male, specifically fathers’ , influence on infant feeding practices [10, 38]. We found some evidence of this in project reports; at least one evaluator cited male decision-making authority in household matters. A study about engaging men to reduce malnutrition in Mozambique found that men were primary influencers for exclusive breastfeeding [39]. The influence of other household actors was not examined in this study but has been shown to influence infant feeding, particularly mothers-in-law (infants’ grandmothers) [40, 41]. The influence of other household actors could confound the association between fathers and EBF. The role and influence of different household actors should be considered when planning EBF promotion and support activities as they may present barriers, or even enabling factors, to achieving the goals of the activities.

Conceptual theory about male engagement in EBF promotion and support is not well developed, and we do not know if the reported male engagement strategies effectively engaged men. We did not attempt an advanced quantitative assessment of the association of male engagement strategies with EBF prevalence changes but merely report those associations as part of our descriptive approach to documenting efforts to engage men in EBF promotion and support in LMIC. National or local campaigns to increase EBF may have contributed to prevalence changes in project areas, although we did not find evidence of such efforts in the project final evaluation reports. Nonetheless, we were careful not to ascribe EBF prevalence increase to these projects’ efforts. All project final evaluation reports were program evaluations and not impact evaluations; therefore, the true extent of the impact of the strategies to engage men is unknown beyond the conclusions drawn in the reports. We did not specifically examine or describe how male engagement strategies could have a detrimental effect on EBF practice nor where male involvement in infant feeding decisions reinforces gender inequality, but these questions merit further research.

If considering implementation on a national scale, it would be important to evaluate the effectiveness of male engagement strategies and conduct multiple tests in different areas to determine if strategies are scalable. More comparative studies and impact evaluations are needed within countries to determine which strategies are most effective at promoting and supporting EBF with different populations. Contextual information about societal dynamics can indicate where and with whom interventions are most efficiently targeted [40]. Specifically, more studies about the effect of male engagement on breastfeeding practices are needed, including formative research about male involvement in decisions regarding infant feeding and women’s desire for male involvement in breastfeeding promotion and support.

## Conclusion

It is important to understand the association between gender norms, male engagement strategies, and EBF prevalence in different contexts. This study augments the literature on this topic by reviewing an array of strategies that have been used in different LMIC. Together, these studies point to the importance of formative research about local gender norms and power structures to inform interventions. With increased emphasis on male involvement in reproductive health care and decisions in different settings, the question of for whom to focus promotion messages and supportive skills merits further research in order to determine the most appropriate and effective way to support EBF.

## Abbreviations

CSHGP: Child Survival and Health Grants Program
EBF: Exclusive breastfeeding
IRB: Institutional Review Board
LAM: Lactational amenorrhea method
LMIC: Low- and middle-income countries
NGO: Nongovernmental organization
USAID: United States Agency for International Development
